# Influencing Factors and New Reference Intervals of Adult Thyroid Volume in Iodine-Sufficient Areas of China

**DOI:** 10.1007/s12011-023-03635-x

**Published:** 2023-05-01

**Authors:** Zheyu Lin, Cihang Lu, Di Teng, Ying Sun, Tingting Liu, Yongze Li, Zhongyan Shan, Weiping Teng, The Thyroid disorders Iodine status, Diabetes Epidemiological Survey Group (TIDE)

**Affiliations:** 1 Department of Endocrinology and Metabolism and the Institute of Endocrinology, The NHC Key Laboratory of Diagnosis and Treatment of Thyroid Diseases, The First Hospital of China Medical University, Shenyang, Liaoning People’s Republic of China 110001; 2 Institute of Endocrinology, Liaoning Provincial Key Laboratory of Endocrine Diseases, The First Hospital of China Medical University, No. 155, Nanjing Bei Street, Shenyang, 110001 People’s Republic of China

**Keywords:** Thyroid volume, Reference interval, Iodine, BSA, TSH, Goiter

## Abstract

The diagnosis of goiter is based on the thyroid volume measured by ultrasound in which iodine plays an important role. The purpose of this study was to evaluate the factors that affect thyroid volume and update the reference range of thyroid volume to provide a new basis for the diagnosis and treatment of goiter. A study population from mainland China was evaluated in the Thyroid disorders, Iodine status and Diabetes Epidemiological (TIDE) study. Thyroid size was measured by ultrasound, and thyroid volume was calculated. A quantile regression model was used to analyze the influence of related factors on the thyroid volume at each percentile. The median urinary iodine concentration (UIC) was 185.54 μg/l. The quantile regression model suggested that body surface area (BSA) and thyroid-stimulating hormone (TSH) were positive and negative factors for thyroid volume, respectively (*p* < 0.01). Excess iodine reduces the median and lower limits of thyroid volume. In iodine-sufficient areas, smoking is no longer a risk factor. The reference value range of thyroid volume stratified by sex was 3.92–19.06 ml for males and 3.1–16.17 ml for females. The maximum reference ranges for stratification by sex and BSA were 4.25–20.98 ml for men and 3.44–18.31 ml for women. The iodine nutrition level of the population in mainland China is sufficient. Iodine has the potential to alter the effect of certain factors on thyroid volume and effect is not regional. The new reference interval of adult thyroid volume based on sex and BSA has been updated, which can be used as a reliable reference for updating the diagnostic criteria of endemic goiter.

**Supplementary Information:**

The online version contains supplementary material available at 10.1007/s12011-023-03635-x.

## Introduction

Iodine is the main trace element that maintains the normal physiological function of the thyroid. The main hormones of the thyroid thyroxine and triiodothyronine rely on iodine for synthesis. Iodine deficiency leads to the enlargement of the thyroid, which leads to iodine-deficient goiter and other related diseases. The diagnosis of goiter mainly depends on normal thyroid volume. In 2004, Zimmermann defined thyroid volume reference interval among children in iodine-sufficient areas as indicators reflecting long-term iodine nutrition [1]. Since the Universal Salt Iodization (USI) policy was launched in 1994, iodine nutrition has been greatly improved. By 2011, the median urinary iodine concentration was 238.6 μg/l [2]. In 2007, China implemented local diagnostic criteria for goiter [3], in which volumes greater than 25 ml and 18 ml were considered goiters for men and women, respectively. This standard has been used for more than 10 years, and the prevention and treatment of iodine deficiency disease (IDD) in China has entered the phase of sustained iodine sufficiency [4]. Given the important effect of iodine on thyroid volume, current standards may be unable to accurately reflect the status of iodine nutrition and thyroid health. In recent years, a number of studies have reported the influencing factors of thyroid volume among children, such as age, sex, iodine nutritional status, body mass index (BMI), and body surface area (BSA), and updated the thyroid volume reference interval. There are few relevant studies among adults. Since age is an important factor that affects thyroid volume, it is necessary to update the adult thyroid volume reference interval. In this study, we used TIDE data to identify new reference intervals and influencing factors for adult thyroid volume in China and to analyze potential sex and BSA specificity.

## Materials and Methods

### Study Population

TIDE data samples were collected from a stratified random sample survey in 31 provinces of China based on sex, age, and rural‒urban ratio, as described in detail in previous studies [5]. The age and sex composition of each community and the urban–rural ratio were determined by using the 2010 national census data of China.

To determine the thyroid volume reference value, the following exclusion criteria were applied: younger than 20 or older than 60 years, thyroid dysfunction, autoimmune thyroid disease, and thyroid nodules.

With the approval of the China Medical University Ethics Committee, all participants signed an informed consent form and agreed to publication. All participants completed a questionnaire that covered demographic characteristics, including sex, age, ethnicity (Han or not), education (below college or college and above), income (over 30,000 Chinese Yuan per year or not), current smoking status, and other information related to thyroid disease, such as family and personal disease history, iodized salt intake, province, and surgical history.

### Data Collection and Laboratory Testing

The standard procedure was used to measure height and weight. Subjects wore light clothing and removed their shoes. The following formula was used to calculate body surface area: BSA = weight ^0.425^ × height ^0.725^ × 0.007184 [6]. After fasting for at least 10 h, venous blood was collected by professionals. From each participant, fasting spot urine samples were also collected. All samples were transported by cold chain to Shenyang, China. Serum and urine samples were stored in a − 80 °C refrigerator before analysis, and relevant biochemical parameters were tested in the Endocrinology Laboratory of China Medical University. UIC was assessed by inductively coupled plasma mass spectrometry (Agilent 7700x; Agilent Technologies). Serum thyroid stimulating hormone (TSH), thyroid peroxidase antibodies (TPOAb), and thyroglobulin antibodies (TgAb) were measured using electrochemiluminescence immunoassay on a Cobas 601 analyzer (Roche Diagnostic, Switzerland). Free thyroxin (FT4) and free triiodothyronine (FT3) levels were measured only if the TSH level was outside the reference range. Detailed detection and sampling methods have been described in our previous studies [7, 8]. Diagnostic criteria for thyroid dysfunction or thyroiditis were provided by the test kit manufacturers. The definition of overt hyperthyroidism was TSH < 0.27 mIU/l, and FT4 > 22.0 pmol/l, or FT3 > 6.8 pmol/l; subclinical hyperthyroidism was TSH < 0.27 mIU/l, and FT4 and FT3 within the normal range (FT4 within 12.0–22.0 pmol/l; FT3 within 3.1–6.8 pmol/l); overt hypothyroidism was TSH > 4.2 mIU/l, and FT4 < 12.0 pmol/l; subclinical hypothyroidism was TSH > 4.2 mIU/l, and FT4 within 12.0–22.0 pmol/l, TPOAb positive was TPOAb > 34 IU/ml; TgAb positive was TgAb > 115 IU/ml; and autoimmune thyroiditis (AIT) was TPOAb > 34 IU/ml or TgAb > 115 IU/ml.

### Ultrasound

Thyroid ultrasound was performed by a specially trained technician using a GE Logiq a100 with a 7.5-mHz linear array transducer with the subject in the supine position. The thyroid was evaluated from three dimensions to measure thyroid size, and the measurement results as well as abnormal ultrasound signs were recorded in detail. The thyroid volume of each lobe was calculated according to the formula *V* (ml) = 0.479 × width (cm) × depth (cm) × length (cm) [9]. Thyroid volume was the sum of both lobes, excluding the isthmus.

### Statistical Analysis

All analyses were performed using R. Continuous variables with a normal distribution are represented by the means ± SDs (standard deviations), while data that did not conform to a normal distribution are represented by the medians (IQRs) (interquartile ranges), and Fisher test was used for analysis. Classification variables are represented by percentages (95% confidence intervals (CIs)), and Chi-square test was used for analysis. Restricted cubic spline (RCS) function curve analysis described the correlation between thyroid volume and each indicator. Quantile regression was used to determine the distribution trend of thyroid volume, and the coefficient of the quantile regression model showed the influence of various factors on the thyroid volume quantile.

## Results

The general characteristics of the reference population are summarized in Table 1. A total of 36,197 subjects were included in this study, including 20,044 males and 16,153 females. The median age of the population was 37 years, and the median UIC was 185.54 μg/l. The median thyroid volume in the population was 8.26 ml, with a greater thyroid volume in men than in women (8.97 ml vs. 7.38 ml) (*p* < 0.01). In addition, BMI (24.58 kg/m^2^ vs. 22.97 kg/m^2^), waist circumference (WC) (86.44 cm vs. 77.81 cm), and BSA (1.82 kg/m^2^ vs. 1.59 kg/m^2^) were higher among men than among women. TSH (2.20 mIU/l vs. 2.03 mIU/l), TPOAb (9.67 IU/ml vs. 9.56 IU/ml), and TgAb (14.29 IU/ml vs. 13.66 IU/ml) levels were higher among women than among men. The population was mainly of Han ethnicity, and there was no difference in education or urban‒rural distribution. Individuals who smoked were mostly male.

**Table 1:** Baseline information table for the reference population

Characteristics	Value			
	Total	Male	Female	*P*
*n*	36,197	20,044	16,153	
Age	37 (28,46)	38 (28,46)	36 (27,45)	< 0.01
BMI	23.89 ± 0.03	24.58 ± 0.04	22.97 ± 0.04	< 0.01
WC	82.77 ± 0.08	86.44 ± 0.11	77.81 ± 0.11	< 0.01
BSA	1.72 ± 0.0012	1.82 ± 0.0014	1.59 ± 0.0012	< 0.01
Thyvol	8.26 (6.30, 10.77)	8.97 (6.92, 11.55)	7.38 (5.73, 9.78)	< 0.01
TSH	2.10 (1.53, 2.78)	2.03 (1.48, 2.70)	2.20 (1.61, 2.89)	< 0.01
TPOAb	9.60 (6.98, 13.05)	9.56 (6.96, 12.94)	9.67 (7.04, 13.21)	0.02
TgAb	13.93 (10.91, 17.10)	13.66 (10.76, 16.63)	14.29 (11.12, 17.85)	< 0.01
UIC	185.54 (124.60, 272.10)	189.70 (130.37, 275.25)	180.00 (117.20, 267.00)	< 0.01
Education level				0.81
Below college	59.96 (59.06, 60.85)	60.03 (59.08, 60.98)	59.86 (58.82, 60.89)	
College and above	40.04 (39.23, 40.86)	39.97 (39.02, 40.92)	40.14 (39.11, 41.18)	
Location				0.54
Urban	51.52 (50.70, 52.34)	51.71 (50.74, 52.68)	51.26 (50.22, 52.31)	
Rural	48.48 (47.58, 49.38)	48.29 (47.32, 49.26)	48.74 (47.69, 49.78)	
Ethnicity				0.55
Han	95.42 (94.42, 96.41)	95.36 (95.04, 95.67)	95.49 (95.17, 95.82)	
Non-Han	4.58 (4.36, 4.81)	4.64 (4.33, 4.96)	4.51 (4.18, 4.83)	
Smoking status				< 0.01
Current nonsmoker	73.92 (72.96, 74.87)	55.50 (54.54, 56.45)	98.71 (98.52, 98.91)	
Current smoker	26.08 (25.41, 26.75)	44.50 (43.55, 45.46)	1.29 (1.09, 1.48)	

*BMI* body mass index, *WC* waist circumference, *BSA* body surface area, *Thyvol* thyroid volume, *TSH* thyroid-stimulating hormone, *UIC* urinary iodine concentration.

**Fig. 1:**
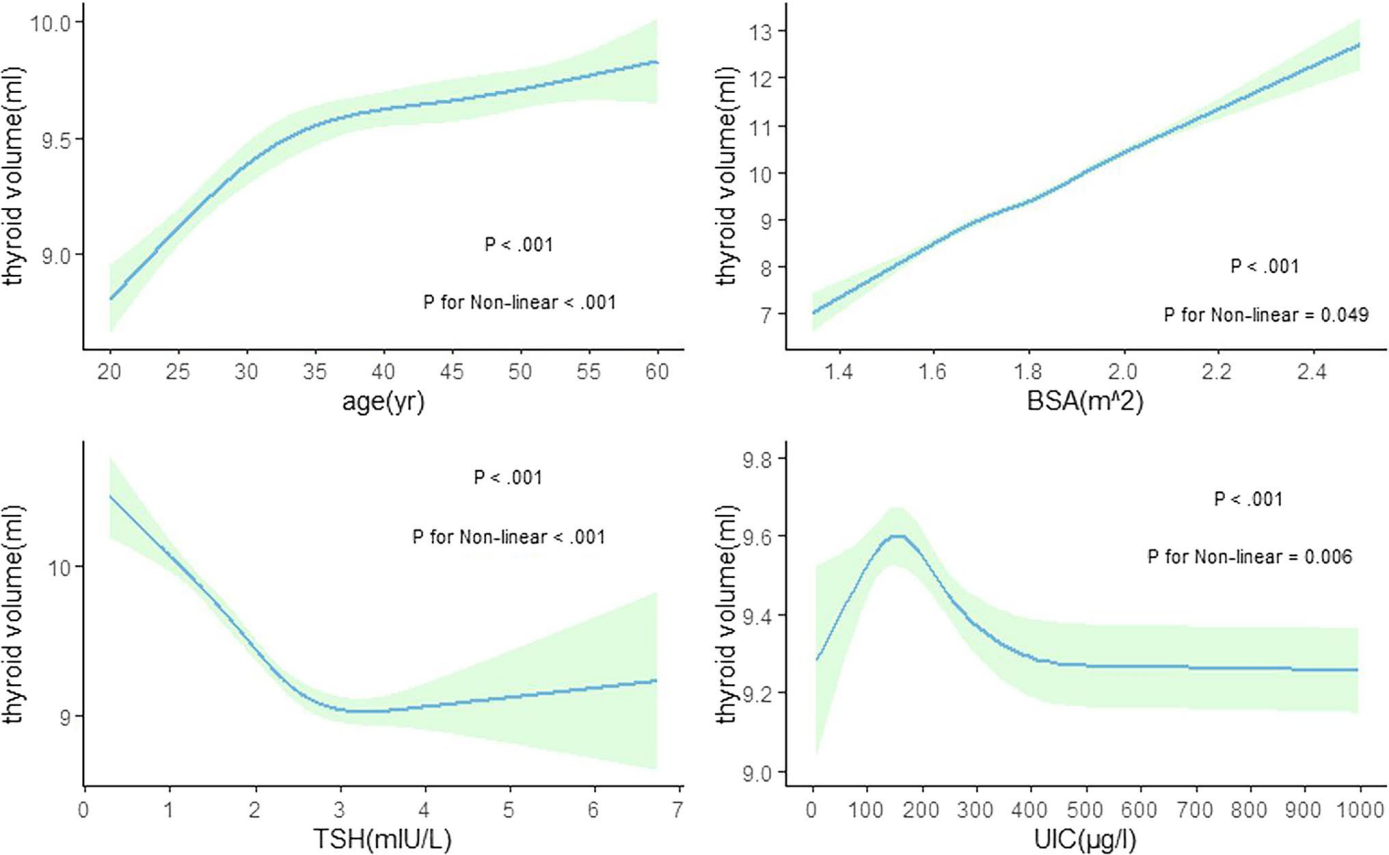
Correlation between thyroid volume and various factors among men

**Fig. 2:**
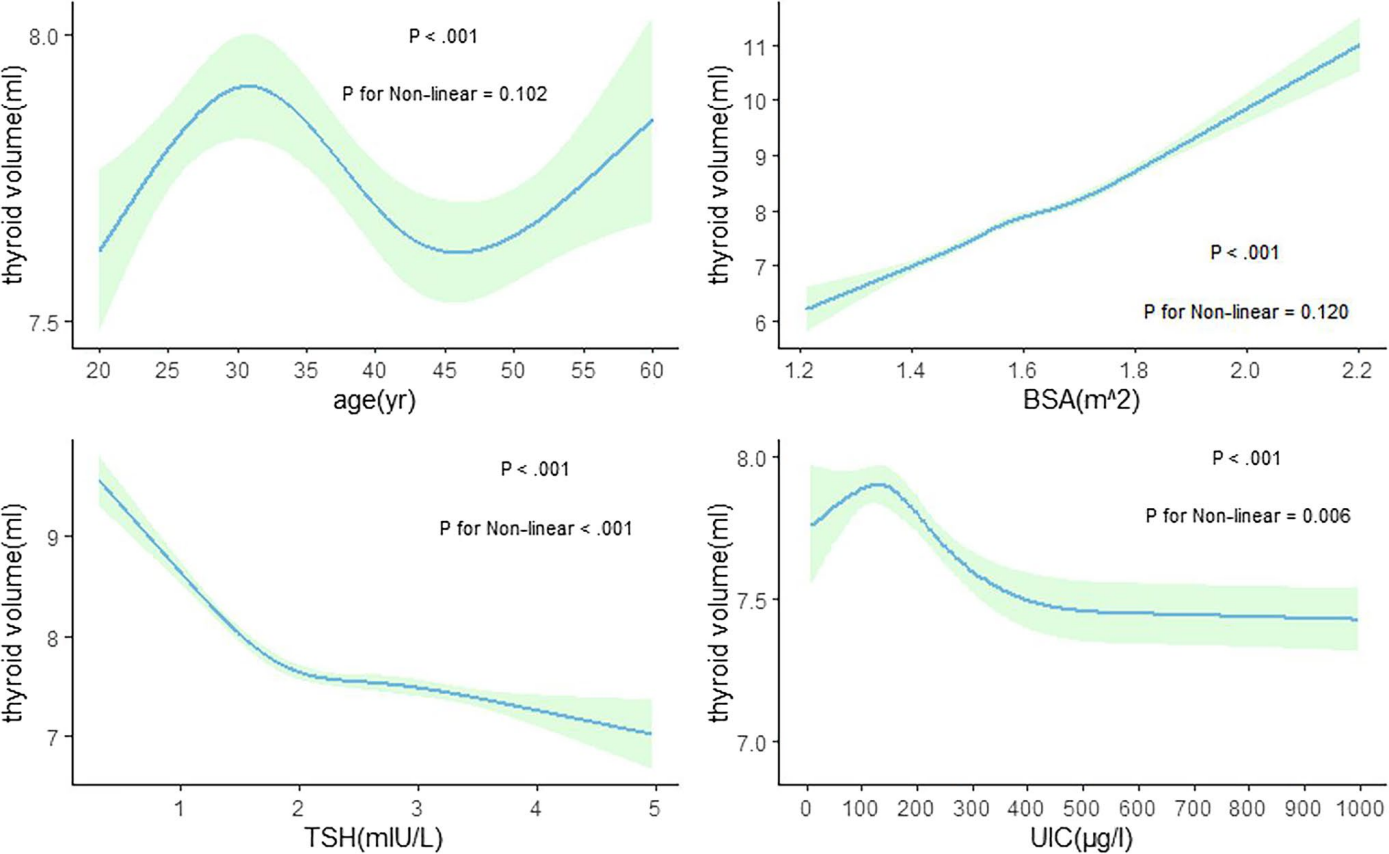
Correlation between thyroid volume and various factors among women

Figures 1 and 2 describe the nonlinear relationship between various factors and thyroid volume for different sexes. Age, BSA, and thyroid volume were positively correlated, while TSH and UIC were negatively correlated.

To establish new reference intervals of adult thyroid volume in iodine-sufficient areas of China, we identified water iodine concentration, iodized salt consumption rates, and salt iodine concentrations in 31 provinces. As shown in Supplementary Table 1, the median concentration of iodine in water was 3.53 μg/l in 31 provinces, and the maximum concentration in Henan was 40.96 μg/l, while the minimum concentration in Sichuan, Shaanxi, and Ningxia was 0 μg/l. The average consumption rate of iodized salt was 96.1% in 31 provinces, of which the highest was 100% in Jiangxi and the lowest was 69.4% in Zhejiang. The median salt iodine concentration was 22.56 μg/l in 31 provinces, with the maximum value of 27.9 μg/l in Chongqing and the minimum value of 0.23 μg/l in Shanghai.

In Table 2, we established a range of reference values for thyroid volume based on sex stratification. The total population reference value range was 3.47–18.28 ml and 3.92–19.06 ml for males and 3.1–16.17 ml for females. The median thyroid volume of the total population was 8.26 ml, with males having a larger volume than females (8.97 ml vs. 7.38 ml), and this trend also existed at the upper and lower limits of the reference value. The difference in thyroid volume at the upper limit (19.06 ml vs. 16.17 ml) was greater than that at the lower limit (3.92 ml vs. 3.1 ml). Moreover, previous studies have proven that thyroid volume is greatly affected by BSA. Therefore, we further stratified the reference values for male and female thyroid volume according to the BSA quartile, as shown in Table 3. The lower limit, median, and upper limit of the reference values of thyroid volume in all population groups increased with the increase in BSA quantiles, and the maximum reference value range based on BSA was 4.25–20.98 ml for males and 3.44–18.31 ml for women.

**Table 2:** Sex-specific thyroid volume reference values

	Quantile (%)	Volume (ml)	CI (2.5–97.5%)
Total	2.5	3.47	3.42–3.54
	50	8.26	8.21–8.33
	97.5	18.28	17.97–18.54
Male	2.5	3.92	3.82–4.06
	50	8.97	8.89–9.05
	97.5	19.06	18.65–19.47
Female	2.5	3.10	3.02–3.18
	50	7.38	7.31–7.45
	97.5	16.17	15.76–16.76

**Table 3:** Sex-specific thyroid volume reference values stratified by BSA

BSA quartile	Thyvol quantile	Volume (ml)	CI (2.5–97.5%)	Volume (ml)	CI (2.5–97.5%)
		Male		Female	
Q1	2.5	3.46	3.24–3.60	2.8	2.59–2.97
	50	8.13	7.98–8.30	6.69	6.56–6.84
	97.5	17.39	15.78–19.60	14.03	13.38–14.70
Q2	2.5	4.13	3.98–4.24	3.02	2.81–3.23
	50	8.71	8.62–8.86	7.17	7.08–7.32
	97.5	17.29	16.78–17.98	14.86	14.32–15.78
Q3	2.5	4.24	3.96–4.43	3.25	3.08–3.44
	50	9.18	9.02–9.35	7.51	7.40–7.63
	97.5	18.84	18.24–19.69	16.07	15.40–17.20
Q4	2.5	4.25	3.85–4.54	3.44	3.30–3.68
	50	9.9	9.69–10.04	8.06	7.93–8.22
	97.5	20.98	20.45–21.86	18.31	17.19–20.57

Male BSA quartile Q1: < 1.70, Q2: 1.70–1.81, Q3: 1.81–1.92, Q4: > 1.92.

Female BSA quartile Q1: < 1.49, Q2: 1.49–1.58, Q3: 1.58–1.67, Q4: > 1.67.

The quantile regression model in Table 4 shows the distribution trend of thyroid volume stratified by sex in the 2.5th (lower limit), 50th (median), and 97.5th (upper limit) centiles. Data in the 25th and 75th centiles are presented in Supplementary Table 2. The median and upper limit thyroid volumes in males increased with age. For each 10-year increase in age for males, the 50th and 97.5th percentiles of thyroid volume increased by 0.12 ml and 0.5 ml, respectively. As shown in Table 2, UIC influenced thyroid volume in both males and females. Stratified analysis of UIC indicated that in the reference population, the lower limit (2.5th percentile) and median (50th percentile) of thyroid volume showed a downward trend with an increase in iodine intake. Compared with the reference group, the lower limit and median thyroid volume in the UIC > 300 µg/l group decreased by 0.28 ml and 0.275 ml and 0.33 ml and 0.39 ml among men and women, respectively. Smoking did not affect thyroid volume in either men or women in our results.

**Table 4:** Quantile regression of the reference population

Thyvol centile	Risk factor	Coefficient	*P* value	95% CI	Coefficient	*P* value	95% CI
		Male			Female		
2.5	(Intercept)	1.24	< 0.01	0.85, 1.63	0.49	0.39	− 0.08, 1.06
	Age	0.00	0.86	0.00, 0.00	− 0.01	0.23	− 0.01, 0.00
	BSA	2.18	0	1.93, 2.33	2.26	0	1.93, 2.60
	TSH	-0.24	0	− 0.30, − 0.19	− 0.27	0	− 0.32, − 0.22
	UIC						
	0–100	0.10	0.38	− 0.01, 0.20	− 0.06	0.60	− 0.17, 0.05
	100–200	Reference	-		Reference	-	
	200–300	0.02	0.85	− 0.11, 0.15	0.08	0.49	− 0.03, 0.19
	300-	-0.28	< 0.01	− 0.37, − 0.20	− 0.28	0.04	− 0.41, − 0.14
	Smoking status						
	Current nonsmoker	Reference	-		Reference	-	
	Current smoker	0.07	0.38	− 0.01, 0.15	0.09	0.75	− 0.20, 0.38
50	(Intercept)	1.27	0.01	0.80, 1.75	1.37	< 0.01	0.91, 1.83
	Age	0.01	< 0.01	0.01, 0.02	0.00	0.76	− 0.01, 0.01
	BSA	4.41	0	4.17, 4.64	4.19	0	3.92, 4.46
	TSH	− 0.35	0	− 0.40, − 0.31	− 0.36	0	− 0.40, − 0.32
	UIC						
	0–100	− 0.08	0.52	− 0.21, 0.05	− 0.04	0.71	− 0.14, 0.07
	100–200	Reference	-		Reference	-	
	200–300	− 0.06	0.49	− 0.16, 0.03	− 0.06	0.50	− 0.15, 0.03
	300–	− 0.33	< 0.01	− 0.44, − 0.23	− 0.39	< 0.01	− 0.50, − 0.29
	Smoking status						
	Current nonsmoker	Reference	-		Reference	-	
	Current smoker	− 0.04	0.65	− 0.12, 0.04	− 0.80	0.26	− 1.07, − 0.54
97.5	(Intercept)	0.25	0.90	− 1.66, 2.16	3.60	0.03	1.94, 5.27
	Age	0.05	< 0.01	0.04, 0.07	0.02	0.14	0.01, 0.04
	BSA	9.30	0	8.40, 10.19	7.58	0	6.61, 8.56
	TSH	− 0.48	< 0.01	− 0.66, − 0.30	− 1.06	0	− 1.20, − 0.91
	UIC						
	0–100	− 0.36	0.39	− 0.77, 0.06	− 0.02	0.94	− 0.33, 0.29
	100–200	Reference	-		Reference	-	
	200–300	− 0.41	0.31	− 0.81, − 0.01	− 0.45	0.24	− 0.83, − 0.07
	300–	− 0.68	0.11	− 1.11, − 0.26	− 0.27	0.45	− 0.63, 0.09
	Smoking status						
	Current nonsmoker	Reference	-		Reference	-	
	Current smoker	− 0.34	0.27	− 0.64, − 0.03	0.26	0.57	− 0.20, 0.71

BSA and TSH, as positive and negative influencing factors among males, affected thyroid volume at each percentile, and this effect gradually increased with increasing percentile. The effect of TPOAb was mainly seen in the 2.5th and 50th centiles, while that of TgAb was only seen in the 2.5th centile. Increased TPOAb and TgAb levels can negatively regulate thyroid volume. In addition, we found that rural area of residence, low income, and low education level were more likely to be the factors influencing the increase in thyroid volume percentile.

The effects of BSA and TSH were the same in women as in men, and the effect of TSH on thyroid volume was much greater in women than in men at the 97.5th percentile, with each unit decrease in TSH resulting in a 1.06 ml increase in thyroid volume. In contrast to men, TPOAb negatively regulated thyroid volume at each percentile, and the effect gradually increased. In addition, rural residence and low income were more likely to be contributing factors to increased thyroid volume in women. Overall, the effect on the upper thyroid volume reference value was greater here, and there was a clear sex difference, with females exhibiting greater sensitivity to TSH and TPOAb.

## Discussion

Iodine status is closely related to thyroid volume, and iodine deficiency leads to thyroid enlargement. The study population was iodine-sufficient with a median urinary iodine level of 185.54 μg/l, which is similar to the results previously reported in Zhejiang, China (178.30 μg/l) [10] and Germany (183.00 μg/l) [11] and higher than those reported in Spain (154.23 μg/l) [12], Tianjin, China (138 μg/l) [13], and Turkey (135 μg/l) [14]. The diagnostic criteria for endemic goiter published in China in 2007 were based on the European iodine-deficient population in 1993 [15]. China is an area with environmental iodine deficiency, and after more than 20 years of salt iodization, iodine nutritional status has changed considerably. Since the implementation of the salt iodization policy in Denmark, the thyroid goiter rate and the volume of the thyroid gland have decreased in all age groups, particularly in areas with moderate iodine deficiency [16]. At the same time, previous studies suggest that the median value of thyroid volume is universal in different areas where iodine is sufficient [13]. Therefore, we propose a new reference value standard for thyroid volume. The reference range assessment required a normal population cohort, and for normal thyroid volume, we selected patients younger than 60 years without thyroid abnormalities, autoimmune thyroid disease, and thyroid nodules. Overall, we are confident that our assessment cohort is reliable and representative.

Many recent studies have evaluated thyroid volume in different regions, and no general sex difference has been found in studies among children. A study in areas with high iodine levels in China found that although age, sex, and BSA can affect thyroid volume in children, when stratified by age, this age-related difference only occurred in the 8-to-9-year-old group. However, among adults, sex has always affected thyroid volume [17, 18]. This may be due to the anatomically larger thyroid and higher lean body mass in males [17] as well as different hormone secretion levels in adulthood. The size difference caused by body weight also exists in other parenchymal organs. Studies have confirmed that the amount of thyroid hormone required by parenchymal organs and muscles may be greater than that required by adipose tissue, so lean body mass determines thyroid volume to a certain extent. In our study, the average thyroid volume in the population was 8.94 ml, with males having a greater volume than females (9.65 ml vs. 8.00 ml), which was similar to the results reported in Spain [12](9.87 ml and 6.57 ml) and among nonpregnant women in Egypt [19](7.9 ml), greater than those reported in Sudan [20](6.69 and 5.78 ml) and Cuba [21](7.3 and 6.4 ml), and smaller than those reported in Turkey [14] (14.53 and 12.09 ml) and the Netherlands (12.7 and 8.7 mL) [17]. The iodine nutrient level, sample size and composition, and geographical location were the main reasons for these differences [20].

Thyroid volume is closely related to anthropometric indexes, and the effect of BSA on thyroid volume has been widely confirmed [10, 18]. In our study, both male and female thyroid volumes were strongly correlated with BSA, and the quantile regression model coefficients for males and females reached 9.3 and 7.5, respectively, at the upper limit of the reference value. BSA could be used as the main standard to evaluate thyroid volume. Our study also found a sex difference in the influence of aging on thyroid volume. The positive age-moderating effect on thyroid volume with the increase in percentile was gradually enhanced among men, which was not significant compared with other factors, whereas there was no such trend among women. Multiple studies have found inconsistent results on the effect of age on thyroid volume. For children, the results of Chen [22] found that age was only a strong predictor of median thyroid volume. An [18] found that only 44% of changes in TVOL were caused by changes in age and BSA. The findings of Guo among adults were consistent with ours, and the effect of age on thyroid volume was also not significant after adjusting for relevant factors [13]. Vejbjerg found that thyroid volume increased with age among women younger than 60 years [16]. The study also showed an age specificity. After 4 years of salt iodization, thyroid volume was normalized in the younger group, while the thyroid goiter rate remained high in the group aged over 40 years. This shows the relationship between age and iodine and thyroid volume, suggesting that it may take years or even generations after long-term exposure to iodine deficiency to normalize thyroid volume in the population. However, a French study concluded that thyroid volume in women (35–60 years old) was negatively correlated with age [23]. Their explanation for this is that aging causes a loss of lean body mass. We believe that age-based bias may also be a contributing factor. A possible explanation for this phenomenon is that the effect of age on thyroid volume is regulated by multiple factors [24, 25] and plays a role by influencing other more important factors related to it.

In addition to growth, development, and genetic characteristics, thyroid volume is also affected by environmental factors, such as different iodine intake [25, 26]. The relationship between iodine and thyroid volume has been confirmed, and excessive iodine can lead to hypothyroidism or hyperthyroidism [27, 28]. In addition, various studies have demonstrated different cutoff values for goiter caused by iodine excess [29, 30]. Zimmerman reported that a UIC > 500 μg/l leads to increased thyroid volume in children aged 6 to 12 years. A UIC > 900 μg/l was associated with a goiter prevalence of more than 10% in northwest Jiangsu, China. Iodine deficiency can lead to goiter, and iodine supplementation in iodine-deficient areas can reduce thyroid volume. In the Danish iodine supplement cohort, the median thyroid volume in men and women with mild iodine deficiency decreased from 15.8 ml and 12.7 ml to 14.1 ml and 11.8 ml, respectively, and the median thyroid volume in men and women with moderate iodine deficiency decreased from 19.5 and 14.2 ml to 16.9 and 12.3 ml, respectively [16]. In our study, increased urinary iodine levels (UIC > 300 μg/l) in iodine-abundant areas reduced thyroid volume in each percentile. This result is consistent with previous results from Jiangsu, Japan, and Brazil [31–33]. Japanese researchers found that the thyroid volume of 8-year-old boys with urinary iodine levels greater than 300 μg/l was smaller than that of boys with urinary iodine levels lower than 300 μg/l. The results of the Jiangsu study indicated that after the elimination of IDDs, the median thyroid volume continued to decline with increasing UIC, and the cutoff point was approximately 300 μg/l. The average thyroid volume of children with a UIC > 300 μg/l was significantly increased, but the average thyroid volume was much smaller than that of the UIC < 300 μg/l group. Many studies have reported the relationship between smoking and thyroid volume [34–36]. In these studies, cigarette smoking was found to increase the size of the thyroid gland, possibly due to thiocyanate competitive inhibition of thyroid iodine uptake [37]. The French study confirmed that in iodine-deficient areas, the thyroid volume of smokers was still larger than that of nonsmokers even after quitting smoking, indicating the persistence of thyroid damage caused by smoking. However, multiple studies have confirmed that smoking in iodine-sufficient areas is not associated with thyroid volume [37–39]. Vejbjerg’s study found that the proportion of goiters caused by smoking was 39.3% before iodine supplementation and 24.2% after iodine supplementation. The urinary iodine-thiocyanate ratio was lower among subjects with goiters, and the competitive inhibitory effect of thiocyanate was weakened by increased iodine intake. This suggests that the effect of smoking on thyroid volume is reduced in areas with moderate iodine sufficiency and that four years of salt iodization has reduced smoking-induced thyroid enlargement [40]. In our study, there was no significant correlation between smoking and thyroid volume after 20 years of salt iodization, which may be related to long-term iodine sufficiency after iodization. This iodine-induced reversal was also seen in studies of serum selenium. In iodine deficiency, serum selenium was significantly negatively associated with goiter in China and Denmark. After adequate iodine supplementation, serum selenium is no longer a risk factor for thyroid volume [41, 42]. This suggests that iodine is important for thyroid volume and that this effect can be generalized to all regions.

We also found a significant negative correlation between TSH and thyroid volume, with the greatest effect on the upper thyroid volume limit in women. This is consistent with the results of several recent studies [12, 13, 23]. The French study also found a positive regulatory effect of FT4 on thyroid volume, while several earlier studies did not find a correlation between TSH and thyroid volume [17, 43, 44]. We speculate that iodine levels may have contributed to the difference in results, with Germany being iodine-deficient at the time and Sweden being iodine-sufficient. The thyroid volume and thyroglobulin (Tg) levels in Germany were higher than those in Sweden, while TSH levels were lower. This suggests that a variety of growth factors and environmental factors may influence the effect of TSH on thyroid growth. The increase in iodine intake in the population is positively correlated with TSH levels, and different iodine nutrient levels may affect the role of TSH in thyroid volume.

To our knowledge, this is the largest cohort to study the reference value of adult thyroid volume, covering all 31 provinces in mainland China, which is regional and representative. Our study has several limitations. As a cross-sectional study, the influence of iodine on thyroid volume over time could not be determined, and the effect of iodine deficiency on thyroid volume and the alleviation of the cumulative effect of iodine supplementation are worthy of attention. And Tg, as a sensitive indicator reflecting iodine nutrition, was not measured.

In conclusion, our study showed that thyroid volume also changed after iodine nutrition level changes in China, and age, BSA, UIC, and TSH are factors that affect thyroid volume. Iodine may interact with many factors and affect thyroid volume in both direct and indirect ways. Our new reference interval for adult thyroid volume could be used as a reliable reference for updating the diagnostic criteria for endemic goiter.

### Supplementary Information

Below is the link to the electronic supplementary material.

Supplementary file1 (DOCX 36 KB)

## Data Availability

The datasets for this study are not publicly available but are available from the corresponding author for reasonable reasons. With the approval of the China Medical University Ethics Committee, all participants signed an informed consent form and agreed to publication. All the authors approved the final version before submission and declare no potential competing interests.

## Abbreviations

TIDE: Thyroid disorders, Iodine status and Diabetes Epidemiological
UIC: Median urinary iodine concentration
BSA: Body surface area
TSH: Thyroid-stimulating hormone
TPOAb: Thyroid peroxidase antibody
TgAb: Thyroglobulin antibody
USI: Universal Salt Iodization
IDD: Iodine deficiency disease
BMI: Body mass index
RCS: Restricted cubic spline
Tg: Thyroglobulin
