# Supplementary material for: Influencing Factors and New Reference Intervals of Adult Thyroid Volume in Iodine-Sufficient Areas of China
Source: Biol Trace Elem Res. 2023 May 1;201(12):5652–61. doi: 10.1007/s12011-023-03635-x (PMC10620313; doi:10.1007/s12011-023-03635-x)
Supplement: Supplementary file 1 — Supplementary file1 (DOCX 36 KB) [file 12011_2023_3635_MOESM1_ESM.docx]

Supplementary table 1 Iodine status in 31 provinces of China

| province | water iodine concentration (μg/L) | iodized salt coverage | salt iodine concentration (μg/L) |
| --- | --- | --- | --- |
| Guangdong | 6.07(5.35-7.17) | 98.7% |  |
| Fujian | 2.85(2.43-3.43) | 93.3% | 19.44(4.36-20.8) |
| Shandong | 5.97(4.78-7.23) | 96.1% | 21.79(10.78-24.36) |
| Tibet | 0.82(0-5.19) | 98.5% | 25.76(22.53-26.85) |
| Xinjiang | 14.57(1.93-20.98) | 99.9% |  |
| Liaoning | 12.73(8.66-33.74) | 98.1% | 22.3(19.57-24.95) |
| Beijing | 2.35(0.75-2.80) | 96.9% | 21.64(21.01-24.05) |
| Hainan | 7.30(4.44-9.63) | 98.3% | 24.46(9.75-27.8) |
| Guangxi | 2.16(1.56-3.01) | 99.5% | 19.34(17.72-21.6) |
| Heilongjiang | 2.46(0.29-6.18) | 98.3% |  |
| Zhejiang | 2.85(0-16.84) | 69.4% | 16.74(0-18.57) |
| Shanghai | 14.31(13.32-18.52) | 81.7% | 0.23(0.02-24.11) |
| Tianjin | 9.27(7.53-11.67) | 81.9% |  |
| Sichuan | 0(0-0) | 97.7% | 25.49(18.45-27.83) |
| Hunan | 19.62(17.22,47.11) | 96.7% | 25.26(23.24-25.69) |
| Jiangxi | 2.99(0-5.32) | 100% |  |
| Yunnan | 1.72(1.33-2.72) | 99.6% | 22.21(20.23-25.75) |
| Jilin |  | 97.5% |  |
| Hubei | 2.39(0-3.04) | 98% |  |
| Chongqing |  | 99.2% | 27.9(23.81-35.4) |
| Guizhou | 0.44(0.21-1.77) | 99.6% |  |
| Hebei | 15.12(8.85-21.42) | 95.7% | 25.04(19.56-28.51) |
| Neimeng | 1.22(0-3.31) | 96.4% |  |
| Anhui |  | 99.5% |  |
| Shanxi | 5.36(3.79-17.80) | 97% |  |
| Qinghai | 2.1291.42-2.51） | 99.4% |  |
| Shaanxi | 0(0-0.06) | 97.9% | 27.24(25.19-28.57) |
| Henan | 40.96(20.13-42.14) | 98.6% | 22.12(20.35-25.41) |
| Gansu | 1.82(1.33-12.87) | 99.8% | 21.06(10.25-25.08) |
| Ningxia | 0(0-7.13) | 98.4% |  |
| Jiangsu |  | 99.4% | 20.44(17.63-21.81) |
| Total | 3.53(0.6-9.68) | 96.1% | 22.56(18.65-25.71) |

Supplementary table 2 Quantile regression of the reference population

| **Thyvol Centile** | **Risk Factor** | **Coeffificient** | **P value** | **95% CI** | **Coeffificient** | **P value** | **95% CI** |
| --- | --- | --- | --- | --- | --- | --- | --- |
|  |  | Male |  |  | Female |  |  |
| **2.5** | **(Intercept)** | 1.23989 | 0.00136 | 0.85276，1.62702 | 0.49125 | 0.38967 | -0.07981，1.06231 |
|  | **Age** | 0.00061 | 0.85546 | -0.00275，0.00397 | -0.00572 | 0.22836 | -0.01047，-0.00097 |
|  | **BSA** | 2.12791 | 0 | 1.92876，2.32706 | 2.2633 | 0 | 1.93075，2.59585 |
|  | **TSH** | -0.24326 | 0 | -0.29509，-0.19143 | -0.26698 | 0 | -0.31512，-0.21884 |
|  | **TPOAb** | -0.02485 | 0.00006 | -0.03104，-0.01866 | -0.02749 | 0.00117 | -0.03595，-0.01903 |
|  | **TgAb** | -0.00822 | 0.03892 | -0.0122，-0.00424 | 0.00957 | 0.03943 | 0.00493，0.01421 |
|  | **UIC** |  |  |  |  |  |  |
|  | 0-100 | 0.09515 | 0.37668 | -0.01248，0.20278 | -0.05921 | 0.59836 | -0.17161，0.05319 |
|  | 100-200 | reference | - |  | reference | - |  |
|  | 200-300 | 0.0241 | 0.85379 | -0.10667，0.15487 | 0.07702 | 0.49038 | -0.03465，0.18869 |
|  | 300- | -0.2848 | 0.00135 | -0.37363，-0.19597 | -0.27589 | 0.03804 | -0.40888，-0.1429 |
|  | **Smoke** |  |  |  |  |  |  |
|  | Current non-smoker | reference | - |  | reference | - |  |
|  | Current smoker | 0.06811 | 0.37918 | -0.00934，0.14556 | 0.09095 | 0.75223 | -0.19715，0.37905 |
|  | **Location** |  |  |  |  |  |  |
|  | Urabn | reference | - |  | reference | - |  |
|  | Rural | 0.15696 | 0.02316 | 0.08785，0.22607 | 0.13123 | 0.17105 | 0.03536，0.2271 |
|  | **Income** |  |  |  |  |  |  |
|  | <30000￥ | reference | - |  | reference | - |  |
|  | ≥30000￥ | -0.30239 | 0.00007 | -0.37832，-0.22646 | -0.03878 | 0.67728 | -0.13196，0.0544 |
|  | **Education** |  |  |  |  |  |  |
|  | Below college | reference | - |  | reference | - |  |
|  | college and above | -0.00094 | 0.99113 | -0.08518，0.0833 | 0.18006 | 0.08173 | 0.07663，0.28349 |
| **25** | **(Intercept)** | 1.16478 | 0.0079 | 0.72633，1.60323 | 1.14172 | 0.0058 | 0.72796，1.55548 |
|  | **Age** | 0.01008 | 0.00567 | 0.00643，0.01373 | -0.00126 | 0.71916 | -0.00476，0.00224 |
|  | **BSA** | 3.5431 | 0 | 3.3259，3.7603 | 3.36157 | 0 | 3.11124，3.6119 |
|  | **TSH** | -0.27011 | 0 | -0.31245，-0.22777 | -0.30527 | 0 | -0.341，-0.26954 |
|  | **TPOAb** | -0.03589 | 0 | -0.04361，-0.02817 | -0.03358 | 0 | -0.03892，-0.02824 |
|  | **TgAb** | -0.00721 | 0.11407 | -0.01178，-0.00264 | 0.00644 | 0.00107 | 0.00447，0.00841 |
|  | **UIC** |  |  |  |  |  |  |
|  | 0-100 | -0.19205 | 0.12408 | -0.31693，-0.06717 | 0.02755 | 0.73468 | -0.05374，0.10884 |
|  | 100-200 | reference | - |  | reference | - |  |
|  | 200-300 | -0.06048 | 0.47635 | -0.1454，0.02444 | 0.00095 | 0.99069 | -0.08022，0.08212 |
|  | 300- | -0.42011 | 0.00002 | -0.51805，-0.32217 | -0.24622 | 0.00932 | -0.3409，-0.15154 |
|  | **Smoke** |  |  |  |  |  |  |
|  | Current non-smoker | reference | - |  | reference | - |  |
|  | Current smoker | 0.01121 | 0.87802 | -0.06184，0.08426 | -0.80041 | 0.00551 | -1.08877，-0.51205 |
|  | **Location** |  |  |  |  |  |  |
|  | Urabn | reference | - |  | reference | - |  |
|  | Rural | 0.44584 | 0 | 0.36958，0.5221 | 0.43465 | 0 | 0.36628，0.50302 |
|  | **Income** |  |  |  |  |  |  |
|  | <30000￥ | reference | - |  | reference | - |  |
|  | ≥30000￥ | 0.11071 | 0.15084 | 0.03365，0.18777 | 0.07999 | 0.21875 | 0.01495，0.14503 |
|  | **Education** |  |  |  |  |  |  |
|  | Below college | reference | - |  | reference | - |  |
|  | College and above | -0.06088 | 0.48028 | -0.14713，0.02537 | 0.17803 | 0.01619 | 0.104，0.25206 |
| **50** | **(Intercept)** | 1.27377 | 0.00769 | 0.79594，1.7516 | 1.37168 | 0.00306 | 0.90862，1.83474 |
|  | **Age** | 0.01245 | 0.00192 | 0.00844，0.01646 | 0.00117 | 0.76093 | -0.00268，0.00502 |
|  | **BSA** | 4.4092 | 0 | 4.17418，4.64422 | 4.18694 | 0 | 3.91641，4.45747 |
|  | **TSH** | -0.35419 | 0 | -0.39911，-0.30927 | -0.35726 | 0 | -0.39726，-0.31726 |
|  | **TPOAb** | -0.0293 | 0.00065 | -0.03789，-0.02071 | -0.04053 | 0 | -0.04747，-0.03359 |
|  | **TgAb** | -0.00405 | 0.42774 | -0.00915，0.00105 | 0.00906 | 0.00178 | 0.00616，0.01196 |
|  | **UIC** |  |  |  |  |  |  |
|  | 0-100 | -0.08438 | 0.51809 | -0.21493，0.04617 | -0.03864 | 0.71017 | -0.1426，0.06532 |
|  | 100-200 | reference | - |  | reference | - |  |
|  | 200-300 | -0.06356 | 0.49193 | -0.15605，0.02893 | -0.0604 | 0.4973 | -0.14939，0.02859 |
|  | 300- | -0.33393 | 0.00153 | -0.43931，-0.22855 | -0.39316 | 0.00015 | -0.49681，-0.28951 |
|  | **Smoke** |  |  |  |  |  |  |
|  | Current non-smoker | reference | - |  | reference | - |  |
|  | Current smoker | -0.03604 | 0.64889 | -0.1152，0.04312 | -0.80136 | 0.0026 | -1.06744，-0.53528 |
|  | **Location** |  |  |  |  |  |  |
|  | Urabn | reference | - |  | reference | - |  |
|  | Rural | 0.75515 | 0 | 0.67133，0.83897 | 0.7219 | 0 | 0.6447，0.7991 |
|  | **Income** |  |  |  |  |  |  |
|  | <30000￥ | reference | - |  | reference | - |  |
|  | ≥30000￥ | 0.34882 | 0.00008 | 0.26026,0.43738 | 0.18779 | 0.01223 | 0.11285，0.26273 |
|  | **Education** |  |  |  |  |  |  |
|  | Below college | reference | - |  | reference | - |  |
|  | College and above | -0.19004 | 0.03711 | -0.2812，-0.09888 | 0.07184 | 0.40525 | -0.01447，0.15815 |
| **75** | **(Intercept)** | 0.2588 | 0.67956 | -0.36774，0.88534 | 1.69493 | 0.00904 | 1.0457，2.34416 |
|  | **Age** | 0.01796 | 0.00077 | 0.01262，0.0233 | -0.00046 | 0.93107 | -0.00576，0.00484 |
|  | **BSA** | 6.23042 | 0 | 5.91999，6.54085 | 5.09263 | 0 | 4.70342，5.48184 |
|  | **TSH** | -0.48489 | 0 | -0.53755，-0.43223 | -0.44565 | 0 | -0.50236，-0.38894 |
|  | **TPOAb** | -0.02375 | 0.03779 | -0.03518，-0.01232 | -0.03056 | 0.00318 | -0.04092，-0.0202 |
|  | **TgAb** | -0.00525 | 0.36749 | -0.01107，0.000569999999999999 | 0.0109 | 0.01191 | 0.00657，0.01523 |
|  | **UIC** |  |  |  |  |  |  |
|  | 0-100 | -0.07048 | 0.62028 | -0.21273，0.07177 | 0.08008 | 0.58596 | -0.06694，0.2271 |
|  | 100-200 | reference | - |  | reference | - |  |
|  | 200-300 | -0.29989 | 0.01959 | -0.42836，-0.17142 | 0.02971 | 0.81698 | -0.09865，0.15807 |
|  | 300- | -0.10154 | 0.49716 | -0.25109，0.04801 | -0.39637 | 0.00347 | -0.53198，-0.26076 |
|  | **Smoke** |  |  |  |  |  |  |
|  | Current non-smoker | reference | - |  | reference | - |  |
|  | Current smoker | 0.02294 | 0.83057 | -0.08426，0.13014 | -0.3071 | 0.54537 | -0.81492，0.20072 |
|  | **Location** |  |  |  |  |  |  |
|  | Urabn | reference | - |  | reference | - |  |
|  | Rural | 1.29959 | 0 | 1.18465，1.41453 | 1.25689 | 0 | 1.14979，1.36399 |
|  | **Income** |  |  |  |  |  |  |
|  | <30000￥ | reference | - |  | reference | - |  |
|  | ≥30000￥ | 0.30982 | 0.00624 | 0.19655，0.42309 | 0.30834 | 0.00277 | 0.20529，0.41139 |
|  | **Education** |  |  |  |  |  |  |
|  | Below college | reference | - |  | reference | - |  |
|  | College and above | -0.33046 | 0.00643 | -0.45172，-0.2092 | 0.19064 | 0.11749 | 0.06886，0.31242 |
| **97.5** | **(Intercept)** | 0.24953 | 0.8961 | -1.66133，2.16039 | 3.60484 | 0.03006 | 1.94327，5.26641 |
|  | **Age** | 0.05131 | 0.00127 | 0.03539，0.06723 | 0.02126 | 0.14415 | 0.00671，0.03581 |
|  | **BSA** | 9.29886 | 0 | 8.40409，10.19363 | 7.58325 | 0 | 6.60622，8.56028 |
|  | **TSH** | -0.48386 | 0.00744 | -0.66462，-0.3031 | -1.05622 | 0 | -1.19548，-0.91696 |
|  | **TPOAb** | -0.06612 | 0.11726 | -0.10833，-0.02391 | -0.06411 | 0.05438 | -0.09743，-0.03079 |
|  | **TgAb** | 0.01622 | 0.54576 | -0.01063，0.04307 | 0.01676 | 0.21328 | 0.0033，0.03022 |
|  | **UIC** |  |  |  |  |  |  |
|  | 0-100 | -0.35828 | 0.38783 | -0.77316，0.0566 | -0.02246 | 0.94227 | -0.33259，0.28767 |
|  | 100-200 | reference | - |  | reference | - |  |
|  | 200-300 | -0.40966 | 0.30801 | -0.81151，-0.00781 | -0.45177 | 0.23569 | -0.83274，-0.0708 |
|  | 300- | -0.68321 | 0.10651 | -1.10647，-0.25995 | -0.27335 | 0.44745 | -0.63316，0.08646 |
|  | **Smoke** |  |  |  |  |  |  |
|  | Current non-smoker | reference | - |  | reference | - |  |
|  | Current smoker | -0.33588 | 0.27331 | -0.64248，-0.02928 | 0.25698 | 0.57228 | -0.19809，0.71205 |
|  | **Location** |  |  |  |  |  |  |
|  | Urabn | reference | - |  | reference | - |  |
|  | Rural | 1.95778 | 0 | 1.6466，2.26896 | 1.90512 | 0 | 1.63024，2.18 |
|  | **Income** |  |  |  |  |  |  |
|  | <30000￥ | reference | - |  | reference | - |  |
|  | ≥30000￥ | -0.28615 | 0.01733 | -0.65378，0.08148 | 0.37253 | 0.17564 | 0.09746，0.64759 |
|  | **Education** |  |  |  |  |  |  |
|  | Below college | reference | - |  | reference | - |  |
|  | College and above | -0.78356 | 0.0216 | -1.12461，-0.44251 | -0.00906 | 0.97499 | -0.29814，0.28002 |
